# The TRPC6 inhibitor, larixyl acetate, is effective in protecting against traumatic brain injury-induced systemic endothelial dysfunction

**DOI:** 10.1186/s12974-019-1407-6

**Published:** 2019-01-31

**Authors:** Xingjuan Chen, Natalie N. Taylor-Nguyen, Ashley M. Riley, B. Paul Herring, Fletcher A. White, Alexander G. Obukhov

**Affiliations:** 1 Department of Cellular and Integrative Physiology, Indiana University School of Medicine, Indianapolis, IN 46202 USA; 2 Department of Anesthesia, Indiana University School of Medicine, Indianapolis, IN 46202 USA; 3 Stark Neurosciences Research Institute, Indiana University School of Medicine, Indianapolis, IN 46202 USA

**Keywords:** Traumatic brain injury, TRPC channels, Endothelial dysfunction, Aorta, Mice

## Abstract

**Background:**

The incidence of traumatic brain injuries (TBIs) is on the rise in the USA. Concussions, or mild TBIs without skull fracture, account for about 75% of all TBIs. Mild TBIs (mTBIs) lead to memory and cognitive deficits, headaches, intraocular pressure rises, axonal degeneration, neuroinflammation, and an array of cerebrovascular dysfunctions, including increased vascular permeability and decreased cerebral blood flow. It has been recently reported that besides vascular dysfunction in the cerebral circulation, mTBI may also cause a significant impairment of endothelial function in the systemic circulation, at least within mesenteric microvessels. In this study, we investigated whether mTBI affects endothelial function in aortas and determined the contribution of transient receptor potential canonical (TRPC) channels to modulating mTBI-associated endothelial dysfunction.

**Methods:**

We used a model of closed-head mTBI in C57BL/6, 129S, 129S-C57BL/6-F2 mice, and 129S-TRPC1 and 129S-C57BL/6-TRPC6 knockout mice to determine the effect of mTBI on endothelial function in mouse aortas employing ex vivo isometric tension measurements. Aortic tissue was also analyzed using immunofluorescence and qRT-PCR for TRPC6 expression following mTBI.

**Results:**

We show that in various strains of mice, mTBI induces a pronounced and long-lasting endothelial dysfunction in the aorta. Ablation of TRPC6 protects mice from mTBI-associated aortic endothelial dysfunction, while TRPC1 ablation does not impact brain injury-induced endothelial impairment in the aorta. Consistent with a role of TRPC6 activation following mTBI, we observed improved endothelial function in wild type control mice subjected to mTBI following 7-day in vivo treatment with larixyl acetate, an inhibitor of TRPC6 channels. Conversely, in vitro treatment with the pro-inflammatory endotoxin lipopolysaccharide, which activates endothelial TRPC6 in a Toll-like receptor type 4 (TLR4)-dependent manner, worsened aortic endothelial dysfunction in wild type mice. Lipopolysaccharide treatment in vitro failed to elicit endothelial dysfunction in TRPC6 knockout mice. No change in endothelial TRPC6 expression was observed 7 days following TBI.

**Conclusions:**

These data suggest that TRPC6 activation may be critical for inducing endothelial dysfunction following closed-head mTBI and that pharmacological inhibition of the channel may be a feasible therapeutic strategy for preventing mTBI-associated systemic endothelial dysfunction.

## Background

Traumatic brain injuries (TBIs) are a major public health concern as they are a common cause of death and disability, both in the USA and worldwide. The incidence of TBIs is increasing in the USA [1, 2]. TBIs can occur after any sort of blow or injury to the head, which can happen in many different situations, including vehicle collisions, exercise- and sports-related injuries, military involvement, and violence. Virtually all individuals regardless of geographical location, socioeconomic status, occupation, or age are at risk of suffering a TBI in their lifetime. Worldwide TBI incidence is estimated to be approximately 69 million individuals per year [3].

TBIs can range from mild (Glasgow Coma Score 13–15, unsustained loss of consciousness, amnesia immediately before or after the incident, alterations in mental state at the time of the incidence, headache, or any focal neurological deficits following the incident) to severe (Glasgow Coma Score 3–8, coma, severe memory loss, permanent and disabling motor deficits) [4]. Mild TBIs (mTBI) are caused by non-penetrating physical impacts without skull fracture. Up to 80% of all TBIs are categorized as mTBIs, which less frequently involved hospital stays and follow-up [1]. Reports of mTBI are more prevalent in young children less than the age of 4, males between ages 15–24, and the elderly, particularly females over the age of 65 [1, 2]. There is also a growing body of evidence indicating that repetitive mTBIs can lead to permanent dysfunction, including increased risk of neurodegenerative disorders (amyotrophic lateral sclerosis, chronic traumatic encephalopathy, Parkinson’s, and Alzheimer’s diseases) [5–7] and cardiovascular complications [8–16].

TBI is a pro-inflammatory condition affecting predominantly the brain, but it also known to cause impairments in other organs, such as the eye, lungs, and mesenteric arteries [17, 18]. During closed-head mTBI, sterile immune reaction at the site of injury involves both the resident microglia and peripherally derived inflammatory cells that are recruited to the brain [19]. The inflammatory reaction is important for clearing damaged cells and repair processes in the brain [19]; however, excessive activation of innate immunity produces a storm of cytokines that may leak through the impaired blood-brain barrier to the cerebral and systemic circulations and initiate collateral damage.

Cerebrovascular dysfunction has long been associated with TBI. In addition, there is evidence indicating that TBIs also likely have significant effects on the systemic vasculature [11]. A recent study found that open-head TBI causes significant microvascular endothelial dysfunction in the mesenteric bed, lasting at least 24 h post-injury [18]. Endothelial dysfunction is an established risk factor for cardiovascular disease [20–22], specifically associated with essential hypertension [23], cerebral ischemia [24], and may precipitate vasospasm.

Transient receptor potential canonical (TRPC) proteins are a family of proteins implicated in modulating smooth muscle and endothelial function [25–27]. TRPC proteins form receptor- and store-operated Ca^2+^ permeable, nonspecific cation channels in the plasma membrane. The seven members in the TRPC subfamily (TRPC1-TRPC7) can be stimulated via G-protein-coupled receptors, such as the histamine H1 receptor, the muscarinic M1 receptor, or the α1 adrenoceptor [25, 26, 28–30]. In mouse endothelial cells, the TRPC6 channel can be activated by bacterial lipopolysaccharides (LPS) in a Toll-like receptor type 4 (TLR4)-dependent manner [27].

In this study, we investigated the long-term effects of mTBI on the systemic vasculature. We used a mouse closed-head mTBI model to determine whether mTBI leads to endothelial dysfunction in the aorta utilizing isometric tension measurements and determined the role of the TRPC1 and TRPC6 channels in the pathogenesis of mTBI-induced aortic endothelial dysfunction using commercially available TRPC1 and TRPC6 knockout mouse strains.

## Methods

### Animals

C57BL/6, 129S, 129S-C57BL/6-F2, 129S-TRPC1-KO (Stock # 37347-JAX), and 129S-C57BL/6-TRPC6-KO (Stock # 37345-JAX) mouse strains were purchased from The Jackson Laboratory and/or were bred in house. Both male and female mice were used. All animal procedures were performed in accordance to the NIH guide and were approved by the Indiana University IACUC. The mice were euthanized under isoflurane anesthesia by decapitations.

### Closed-head mild traumatic brain injury

**Fig. 1:**
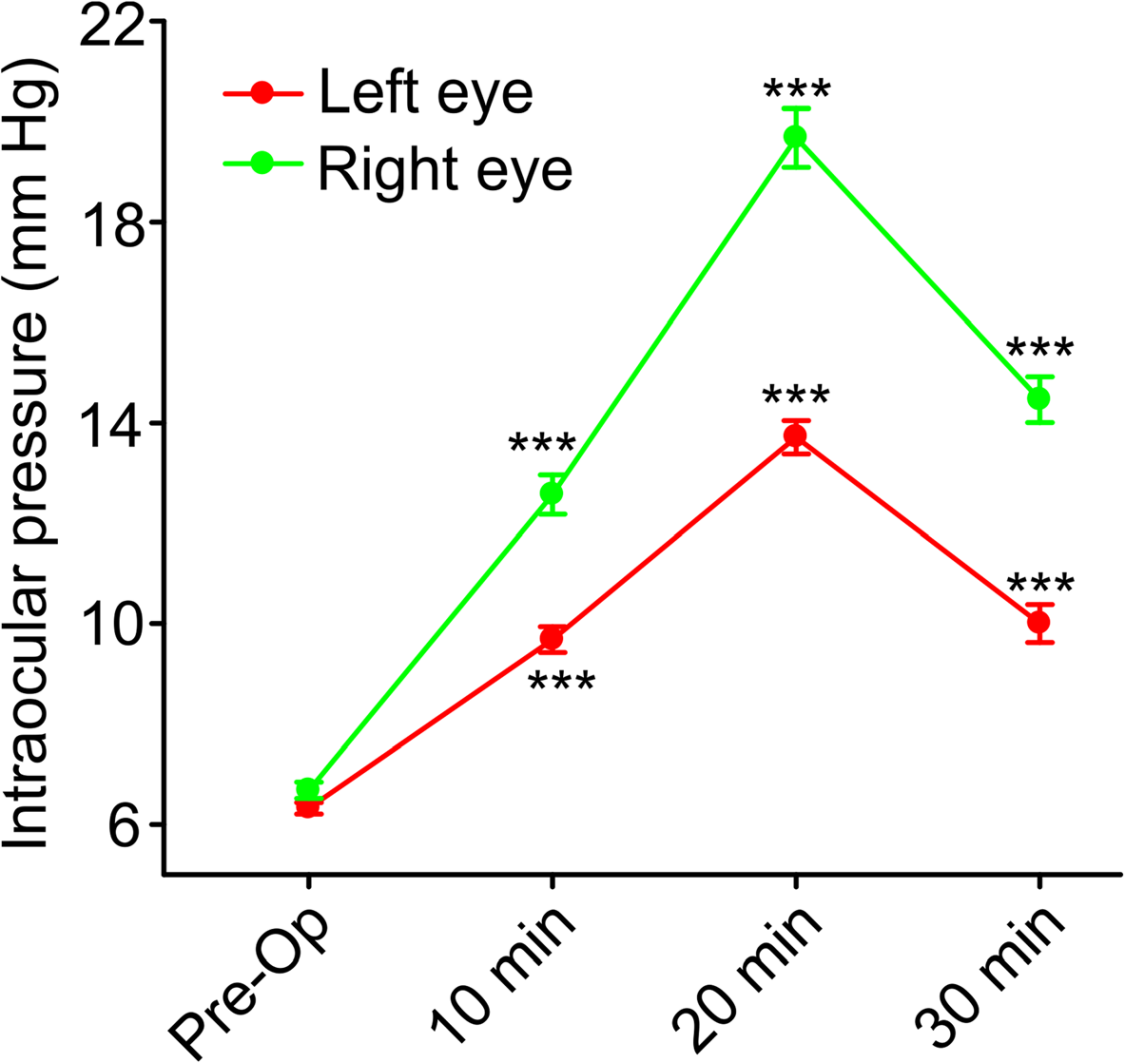
Time course of intraocular pressure (IOP) changes induced by mild TBI. The first reading (Pre-Op) was taken just before the surgery and then 10, 20, and 30 min after inflicting a TBI. None of the mice used for the experiments exhibited evidence of bone fracture. *** stands for *p* < 0.001

Mice were subjected either to a single closed-head TBI using a computer-controlled cortical electromagnetic impactor (Stereotaxic Impactor, Leica Biosystems Inc. Buffalo Grove, IL) or to sham injury as described elsewhere [17, 31]. Briefly, mice were anesthetized by inhalation of 4% isoflurane for induction and 2% isoflurane for maintenance. In addition, a lidocaine (2 mg/kg) and bupivacaine (1 mg/kg) mixture was given locally prior to incision for analgesia. The anesthetized mice were placed in a stereotaxic frame (Kopf Instruments). The top of the head was shaved and a mid-line incision was made from bregma to lambda on the top of the head exposing the skull. An impacting piston with a tip diameter of 3 mm was angled in a way to ensure that its axis was perpendicular to the exposed left parietemporal skull surface. Sham control animals were exposed to anesthesia, subjected to skin incision and skull exposure but was not subjected to the cortical impact. After injury, the incision was sutured and mice were allowed to recover from anesthesia. To confirm the severity of the cortical bone impact and absence of bone fracture, we measured intraocular pressure (IOP) changes in the mouse eye with the Icare TONOLAB (Vantaa, Finland) immediately before and after the injury for up to 30 min (Fig. 1, *n* = 28 mice in each group). Changes in IOP served as a surrogate of changes in brain pressure after the injury event. If the skull bone was fractured, there would be no IOP. In a subset of experiments, these TBI procedures were followed by administration of larixyl acetate (TRPC6 inhibitor) or TAK-252 (TLR4 inhibitor) dissolved in 0.1% DMSO and brought to 1 ml total volume. Drug solution or vehicle were intraperitoneally injected (larixyl acetate, 5 mg/kg; or TAK-242, 10 mg/kg under isoflurane anesthesia) within first 2 h after TBI induction and then on a daily basis for 7 days. In these in vivo experiments, the 7-day post-injury time point has been chosen because a typical hospital stay of a TBI human subject is 7 days (https://www.hcup-us.ahrq.gov/reports/statbriefs/sb27.pdf).

### Assessment of endothelial-dependent vasodilation

Mice were euthanized by decapitations under isoflurane anesthesia. Mouse aortas were isolated and cleaned from the fat and connective tissue in a calcium and magnesium-free phosphate buffer solution (PBS, Lonza). A wire myograph from GlobalTown Microtech., Inc. (Sarasota, FL) was used to monitor the force generated by aortic arch rings. The isometric tension measurements were performed as described elsewhere [32–35]. Briefly, aortic arches were hung on the wires of the wire myograph and were placed into the 5-ml tissue baths filled with the standard Krebs buffer maintained at 37 °C and continuously oxygenated by bubbling a gas mixture of 95% O_2_ and 5% CO_2_ during all of the performed experiments. The preload in all experiments was set to 0.7–1 g. Increasing concentrations of phenylephrine and then acetylcholine was added directly into the tissue baths while the contraction force was measured. SNAP (S-Nitroso-*N*-acetyl-DL-penicillamine, a nitric oxide donor) was used to assess the maximal receptor-independent aortic ring dilations. The analog ring tension data were digitized with a frequency of 20 Hz and recorded on a computer’s hard drive.

### Immunofluorescence staining

Mouse aortas were prepared as described above. Isolated mouse aortas were fixed in 4% paraformaldehyde for 2 h on ice. Tissue was then washed in phosphate buffered saline, and frozen in Tissue-Tek O.C.T Compound (Sakura Finete) using a mixture of dry ice and isopentane. After cryosections (~ 8 μm) were obtained, O.C.T. was removed from tissue samples by a 5–10 min wash in Tris buffered saline (TBS). Samples were permeabilized in 0.2% Triton X-100 in TBS for 5 min and were then washed three times in TBS. After blocking in 5% horse serum in TBS at room temperature for 1 h, sections were incubated at 37° with the TRPC6 antibody (1:100, Alomone lab, ACC-017) and an anti-smooth muscle actin antibody conjugated to Cy3 (1:250; C1698, Sigma). Sections without the TRPC6 primary antibody served as a negative control. TRPC6 immunoreactivity was detected with anti-rabbit Alexa Fluor 647 (1:4000; Jackson ImmunoResearch). Stained samples were mounted in ProLong Gold with DAPI (Invitrogen) and visualized by confocal microscopy (Olympus Fluoview FV1000).

#### qRT-PCR

Total mRNA was isolated from freshly collected mouse aortas using the PureLink mRNA isolation kit (Thermo Fisher Scientific). Five hundred nanograms of RNA was used in reverse transcription reactions (High-Capacity RT-cDNA Kit, Life Technologies). Real-time PCR was performed using Syber Green (Roche). Levels of mRNA expression were normalized to expression of Hprt as an internal control and are expressed relative to the mean values seen in samples from sham control mice. Primers used for PCR were TRPC1 Forward: TCC CAA AGA GCA GAA GGA CTG, TRPC1 Reverse: CAA AGC AGG TGC CAA TGA A; mTRPC6, RT^2^ qPCR assay (PPM04056A, Qiagen).

### Drugs and solutions

All drugs were purchased from Sigma-Aldrich or Cayman Biochemical. The solution composition was as follows. The standard Krebs buffer contained (in millimolar) 130 NaCl, 5 KCl, 2 CaCl_2_, 1.2 NaH_2_PO_4_, 0.56 MgCl_2_, 25 NaHCO_3_, and 5 glucose. The 70 KCl solution contained (in millimolar) 65 NaCl, 70 KCl, 2 CaCl_2_, 1.2 NaH_2_PO_4_, 0.56 MgCl_2_, 25 NaHCO_3_, and 5 glucose.

### Statistical analysis

Sigma plot 12 was used to analyze the aortic ring tension data. Two-way ANOVA followed by a Student-Newman-Keuls post hoc pairwise multiple comparison test was used to compare the tested experimental groups affected by two different factors when the data sets were normally distributed populations with equal variances. The *t* test was used to compare two tested groups. The data sets were considered significantly different if the *p* value was less than 0.05. All data were presented as mean ± standard error (S.E.).

## Results

### Aortas from TBI mice exhibit a pronounced endothelial dysfunction

**Fig. 2:**
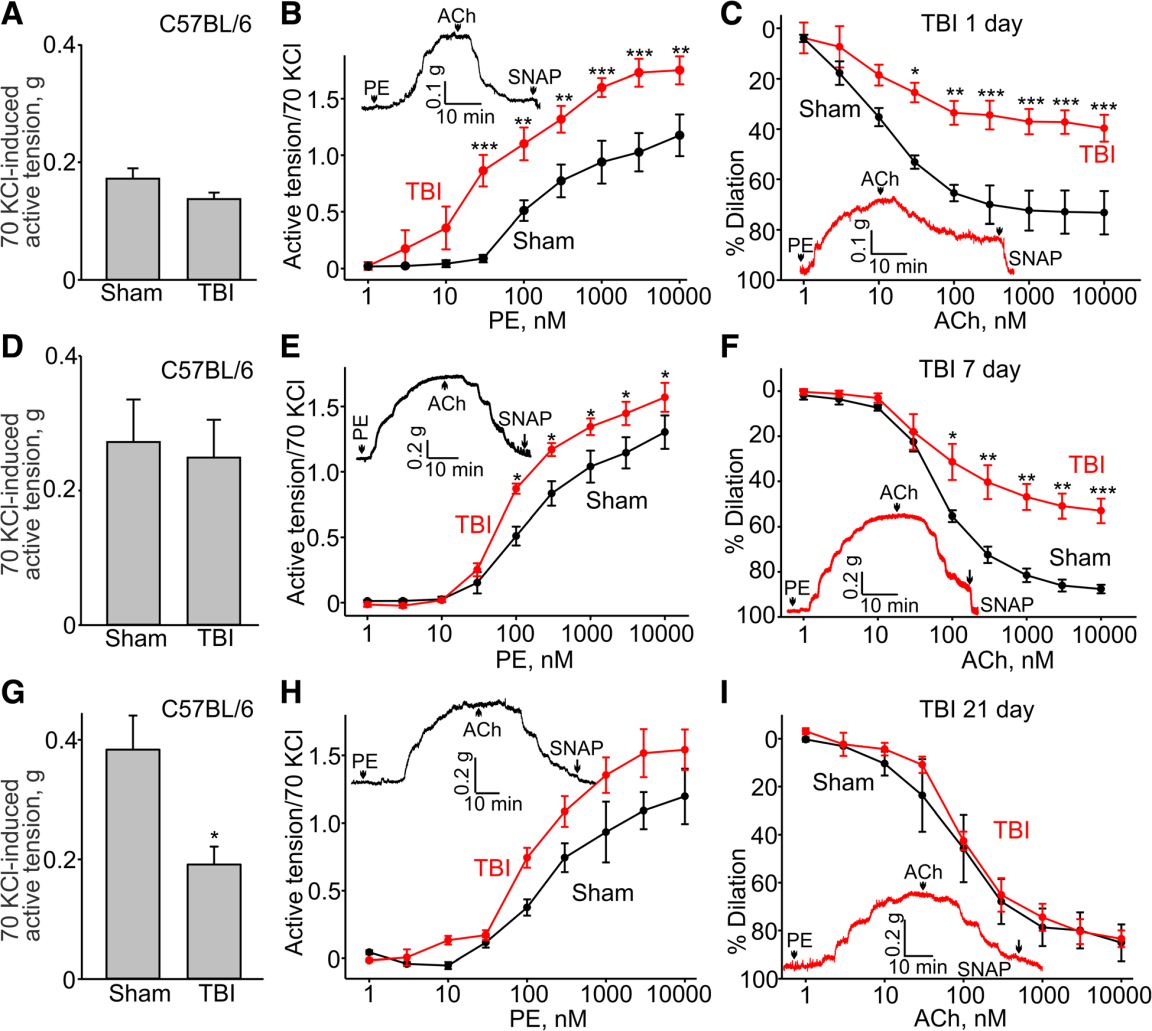
Effect of TBI on reactivity and endothelial function in mouse aortic arches from C57BL/6. **a**, **d**, and **g** Bar graphs comparing the active tensions in C57BL/6 aortic arch rings, respectively, in the presence of 70 mM KCl in sham and TBI mice after 1, 7, and 21 days. **b**, **e**, and **h** Concentration-response relationships for phenylephrine (PE)-induced contractions in aortic arch rings from sham and TBI C57BL/6 mice after 1, 7, and 21 days. **c**, **f**, and **i** Concentration-response relationships for acetylcholine-induced dilation in phenylephrine-precontracted rings from sham and TBI C57BL/6 mice. The insets show the sample traces of active tension changes in aortas from sham (black lines) and TBI (red lines) mice. PE - phenylephrine, ACh - acetylcholine

A major function of the endothelium is to release vasodilatory molecules, such as nitric oxide (NO), which regulates vascular tone. Dysfunction of the endothelium disrupting this regulation increases the risk for developing vascular diseases. It was reported recently that open-head TBIs may cause systemic microvascular endothelial dysfunction, as demonstrated in mesenteric vasculature, at 24 h post-injury. We set out to establish whether closed-head mild TBI causes endothelial dysfunction in the conduit systemic circulation and whether it lasts beyond 24 h post-injury. We first performed isometric tension recordings on rings from aortic arch of C57BL/6 mice subjected to the closed-head mild TBI or sham surgery procedures. Figure 2a shows that mild TBI did not affect the amplitude of maximal KCl-mediated contractions of the aortic arch rings from C57BL/6 mice, but it did increase the amplitude of phenylephrine-induced contractions of the TBI rings compared to sham rings (10 μM phenylephrine-induced active tension normalized to the peak amplitude of 70 mM KCl-stimulated contraction: 1.18 ± 0.14 versus 1.75 ± 0.12 for sham and TBI, respectively, Fig. 2b) and shift the EC_50_ value for phenylephrine to the left in TBI aortas (EC_50_ = 141.5 ± 51.7 nM versus 35.8 ± 21.9 nM for sham and TBI aortas, respectively, Fig. 2b), indicating that the TBI aortas were more sensitive to phenylephrine. We assessed endothelial function by examining the ability of acetylcholine to induce relaxation of aortic rings precontracted with phenylephrine. Compared to sham rings, the TBI rings exhibited significantly reduced acetylcholine-induced dilations (10 μM acetylcholine-induced dilations: 73.3 ± 8.5%, *n* = 3 versus 39.6 ± 5.3%, *n* = 3 for sham and TBI, respectively, Fig. 2c). We next investigated whether TBI-induced changes in vascular reactivity and endothelial function would persist for a longer period of time. We found that 7 days after TBI, 10 μM phenylephrine-induced active tension normalized to the peak amplitude of 70 mM KCl-induced contraction was still greater in TBI than sham mice (1.6 ± 0.11 versus 1.3 ± 0.13 for TBI and sham, respectively, Fig. 2b), though the amplitude of maximal KCl-mediated contractions of the aortic arch rings did not change (0.27 ± 0.06 g versus 0.25 ± 0.07 g for sham and TBI, Fig. 2d). The EC_50_ value for phenylephrine was still shifted to the left in TBI aortas (EC_50_ = 90 ± 13 nM and 159 ± 43 nM for sham and TBI aortas, respectively, Fig. 2e). Remarkably, the ability of acetylcholine to induce relaxation of aortic rings precontracted with phenylephrine was still impaired 7 days post-injury (Fig. 2f). Compared to sham rings, the TBI rings exhibited significantly reduced dilations to 10 μM acetylcholine (87.6 ± 1.9%, *n* = 5 and 53.1 ± 5.4%, *n* = 8 for sham and TBI, respectively, Fig. 2f). However, we found no difference between the TBI and sham groups 21 days post-injury (Fig. 2g, h, and i).

**Fig. 3:**
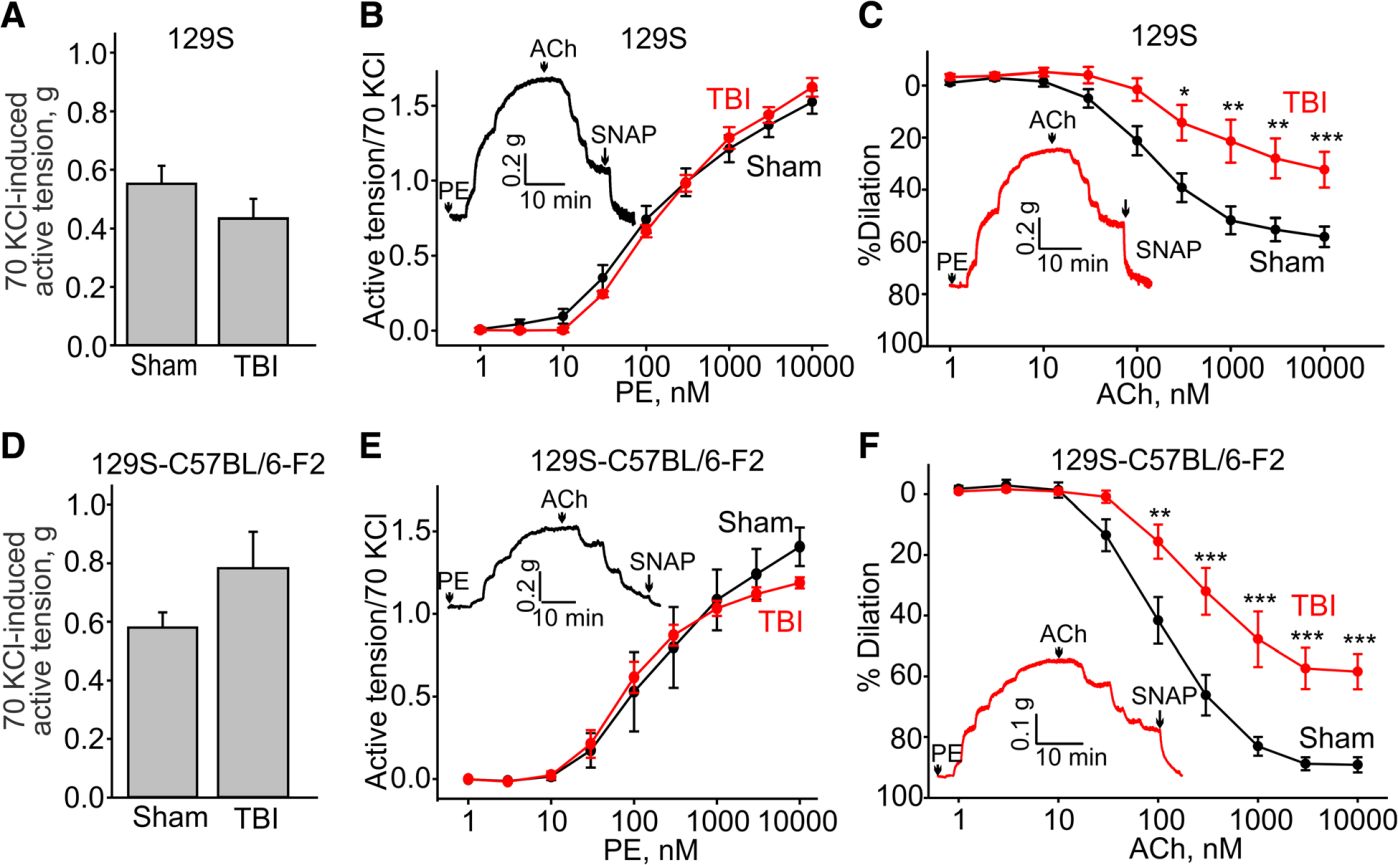
129S and 129S-C57BL/6-F2 mice exhibit similar TBI-associated aortic endothelial dysfunction as the one observed in C57BL/6 mice. **a**, **d** Summary data for the active tensions measured in the presence of 70 mM KCl in aortic arch rings from sham or TBI mice. **b**, **e** Concentration-response relationships for phenylephrine-induced contractions in aortic arch rings from sham and TBI mice. **c**, **f** Concentration-response relationships for acetylcholine-induced dilations in phenylephrine-precontracted rings from sham and TBI 129S-TRPC1 knockout mice. The insets in **b**, **c**, **e**, and **f** show the sample traces of active tension changes in aortas from sham (black lines) and TBI (red lines) mice. PE - phenylephrine, ACh - acetylcholine

To establish the generality of our findings, we also assessed the effect of TBI on the systemic vasculature in mice of two other genetic backgrounds. The amplitude of maximal KCl-induced contractions were similar in both sham and TBI mice on the 129S (0.55 ± 0.06 g versus 0.45 ± 0.07 g, *n* = 9, Fig. 3a) and 129S-C57BL/6-F2 (0.58 ± 0.05 g versus 0.78 ± 0.15 g, *n* = 5, Fig. 3d). The observed increased sensitivity to phenylephrine in the C57BL/6 mice following TBI was not seen in the 129S strain (EC_50_ of 166.1 ± 22.9 nM following TBI compared to EC_50_ of 122.3 ± 33.1 nM in sham mice) or in 129S-C57BL/6-F2 mice (EC_50_ of 94.8 ± 15.2 nM following TBI compared to EC_50_ of 107.6 ± 11 nM in sham mice) (Fig. 3b and e). However, aortic rings from both 129S and 129S-C57BL/6-F2 mice showed pronounced endothelial dysfunction as evidenced by impaired acetylcholine-induced relaxation of phenylephrine-precontracted rings (10 μM acetylcholine-induced dilations: 129S sham 58.0 ± 4.0% versus TBI 32.3 ± 6.8%, Fig. 3c, and 129S-C57BL/6-F2 sham 89.2 ± 2.5% versus TBI 58.4 ± 5.8%, Fig. 3f). Although TBI impaired acetylcholine-induced aortic relaxation in each of the strains of mice, we noted that the average acetylcholine-induced dilation was less pronounced in sham 129S aortic arch rings (10 μM acetylcholine-induced dilation: 58.0 ± 4.0%) compared to that observed in sham aortic arch rings isolated from C57BL/6 mice (10 μM acetylcholine-induced dilation: 89.2 ± 2.5%).

### TRPC1 genetic ablation does not affect TBI-associated endothelial dysfunction

**Fig. 4:**
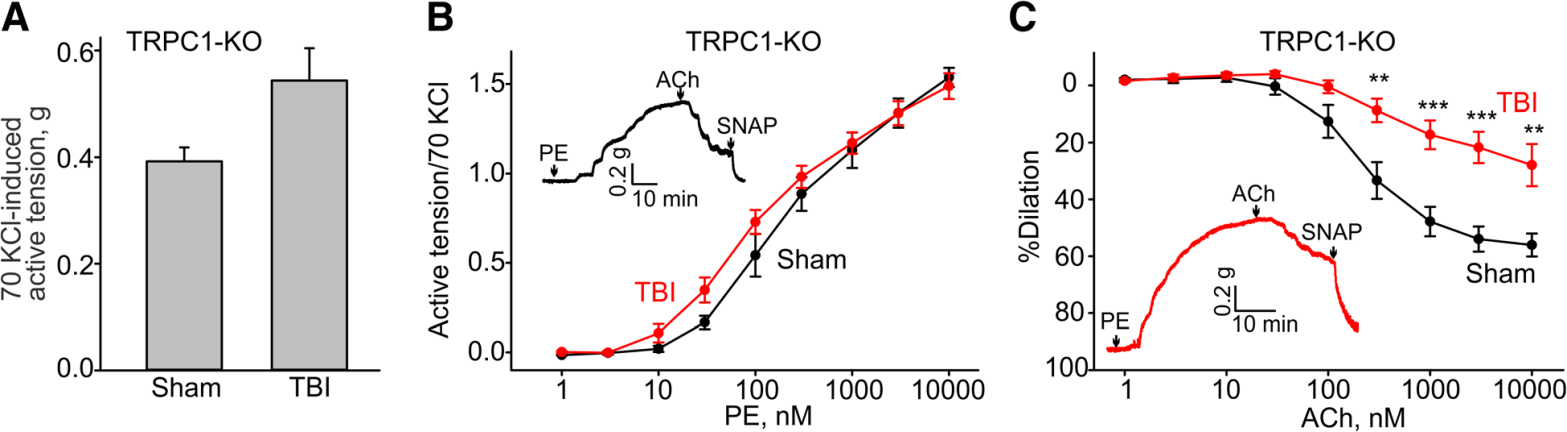
129S-TRPC1 knockout mice exhibit similar TBI-associated aortic endothelial dysfunction as the one observed in the control wild type 129S mice. **a** Summary data for the active tensions measured in the presence of 70 mM KCl in aortic arch rings from sham or TBI TRPC1 knockout mice. **b** Concentration-response relationships for phenylephrine-induced contractions in aortic arch rings from sham and TBI 129S-TRPC1 knockout mice. **c** Concentration-response relationships for acetylcholine-induced dilations in phenylephrine-precontracted rings from sham and TBI 129S-TRPC1 knockout mice. The insets in **b** and **c** show the sample traces of active tension changes in aortas from sham (black lines) and TBI (red lines) mice. PE - phenylephrine, ACh - acetylcholine

TRPC1 and TRPC6 channels are implicated in modulating endothelial function [27]. Therefore, we next assessed whether genetic ablation of either of these channels would affect the TBI-associated endothelial dysfunction. We found that aortic arches from TRPC1 knockout mice (on a 129S background) exhibited the same TBI-mediated endothelial dysfunction as the 129S control mice (10 μM acetylcholine-induced dilation of 56.1 ± 4.1% in sham 129S-TRPC1 KO versus 27.9 ± 7.4% in TBI-129S-TRPC1 knockout mice compared to 58.0 ± 4.0% in sham versus 32.3 ± 6.8% in TBI in 129S control mice; Fig. 4c and Fig. 3c). The average amplitudes of 70 mM KCl-induced contractions in the same sham and TBI aortic rings were not different (0.55 ± 0.06 g, *n* = 9 versus 0.43 ± 0.07 g, *n* = 9 for TBI and sham, respectively, Fig. 4a).

### TRPC6 genetic ablation prevents TBI-associated endothelial dysfunction

**Fig. 5:**
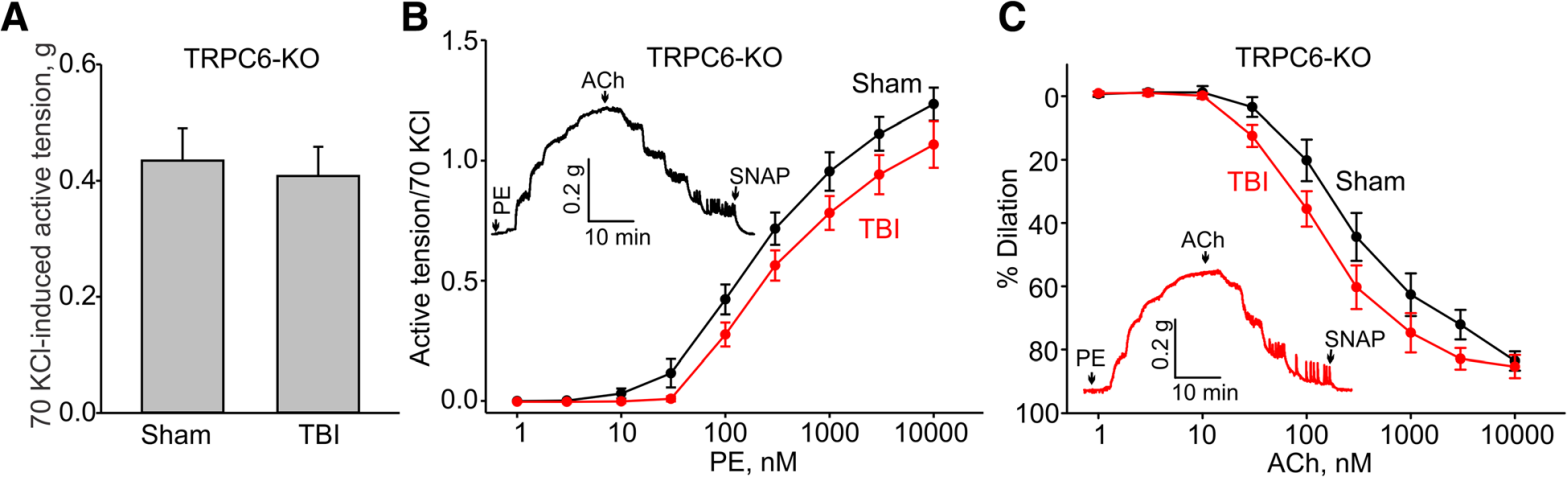
Genetic ablation of TRPC6 in 129S-C57BL/6-F2 mice prevents TBI-associated aortic endothelial dysfunction. **a** Summary data for the active tensions measured in the presence of 70 mM KCl in aortic arch rings from sham or TBI TRPC6 knockout mice. **b** Concentration-response relationships for phenylephrine-induced contractions in aortic arch rings from sham and TBI TRPC6 knockout mice. **c** Concentration-response relationships for acetylcholine-induced contractions in phenylephrine-precontracted rings from sham and TBI TRPC6 knockout mice. The insets in **b** and **c** show the sample traces of active tension changes in aortas from sham (black lines) and TBI (red lines) mice. PE - phenylephrine, ACh - acetylcholine

We next assessed vascular reactivity and endothelial function in the aortic arch of TRPC6 knockout mice on a mixed 129S-C57BL/6-F2 background subjected to TBI or sham procedure. The amplitude of maximal KCl-induced contractions in the TRPC6 mice was not different from that seen in the control 129S-C57BL/6-F2 mice following TBI or in sham mice (Fig. 5a). Phenylephrine-induced contractions had similar EC_50_ values in sham and TBI aortas (EC_50_: 288.6 ± 64.2 nM, *n* = 8 versus 218.8 ± 45.8 nM, *n* = 8, Fig. 5b). Remarkably, we observed that aortic rings from TBI TRPC6 knockout mice exhibited endothelial function that was indistinguishable from that observed in sham TRPC6 knockout animals (10 μM acetylcholine-induced dilation: 83.6 ± 3.0% versus 85.3 ± 3.6%, respectively, Fig. 5c). These data show that loss of TRPC6 protects mice from TBI-induced endothelial dysfunction.

### TRPC6 activation is critical for mediating TBI-associated endothelial dysfunction

**Fig. 6:**
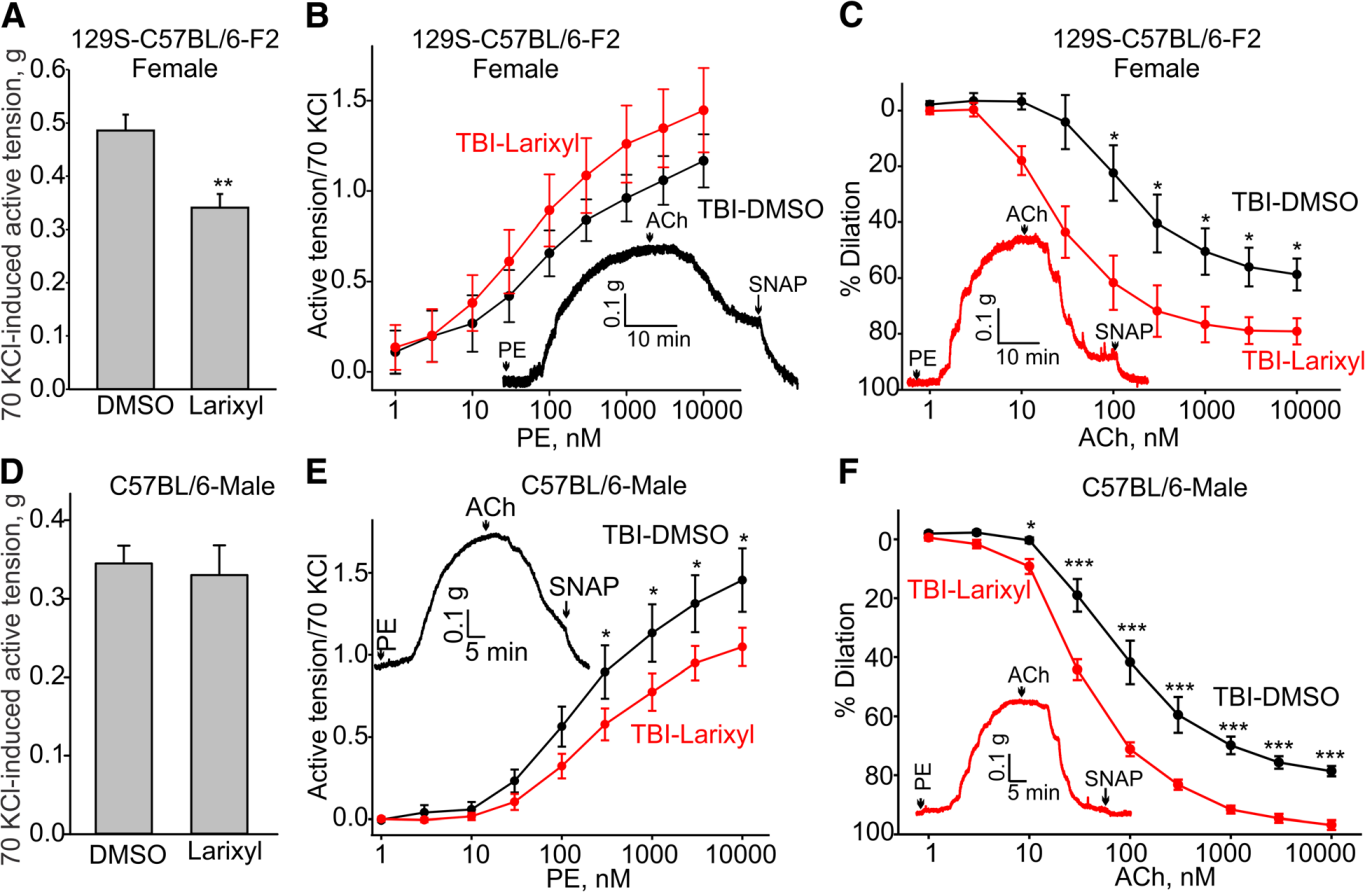
Pharmacological inhibition of TRPC6 in 129S-C57BL/6-F2 and C57BL/6 mice prevented TBI-associated aortic endothelial dysfunction. **a**, **d** Summary data for the active tensions measured in the presence of 70 mM KCl in aortic arch rings from TBI 129S-C57BL/6-F2 or C57BL/6 mice treated in vivo with DMSO or larixyl via intraperitoneal injection at 5 mg/kg/day daily for 7 days. **b** Concentration-response relationships for phenylephrine-induced contractions in aortic arch rings from TBI 129S-C57BL/6-F2 mice treated with DMSO or larixyl acetate in vivo. **c** Concentration-response relationships for acetylcholine-induced contractions in phenylephrine-precontracted rings from sham and TBI 129S-C57BL/6-F2 mice treated with DMSO or larixyl in vivo. **e** Concentration-response relationships for phenylephrine-induced contractions in aortic arch rings from TBI C57BL/6-F2 mice treated with DMSO or larixyl acetate in vivo. **f** Concentration-response relationships for acetylcholine-induced contractions in phenylephrine-precontracted rings from sham and TBI C57BL/6 mice treated with DMSO or larixyl in vivo. The insets in **b**, **c**, **e**, and **f** show the sample traces of active tension changes in aortas from the TBI-DMSO group (black lines) and TBI-larixyl group (red lines) mice. PE - phenylephrine, ACh - acetylcholine

Since TRPC6 knockout mice exhibited no TBI-associated endothelial dysfunction, we next tested whether pharmacological inhibition of the channel would show any therapeutic potential. Larixyl acetate has been identified as a potent and relatively selective inhibitor of TRPC6 [36]. Therefore, we next tested whether larixyl-mediated inhibition of TRPC6 activity in vivo would be efficacious at reducing TBI-associated endothelial dysfunction in 129S-C57BL/6-F2 and C57BL/6 mice subjected to TBI and treated with larixyl acetate. During these experiments, we assessed the effects of larixyl acetate treatment on aortic reactivity (in the presence of 70 mM KCl and 10 μM phenylephrine) and endothelial function 7 days after closed-head TBI with half of the mice receiving either DMSO vehicle control or larixyl acetate. Larixyl acetate or DMSO was given to the mice by intraperitoneal injection immediately after inflicting TBI and then on a daily basis for 7 days. Laryxil acetate treatment did not affect aortic ring sensitivity to 70 mM KCl-induced depolarization in C57BL/6 mice, but significantly decreased it in 129S-C57BL/6-F2 mice (Fig. 6a and d). Conversely, phenylephrine-induced contractions were significantly decreased in larixyl-treated C57BL/6 mice (Fig. 6e), but it was unaffected in 129S-C57BL/6-F2 mice. Importantly, we found that larixyl acetate treatment was very effective in significantly reducing TBI-associated endothelial dysfunction in both female and male mice (Fig. 6). 10 μM  acetylcholine-induced dilations were 58.4 ± 5.8% for DMSO versus 89.2 ± 2.5% for larixyl in 129S-C57BL/6-F2 females (*n* = 4 for DMSO and n = 5 for larixyl) or 78.6 ± 1.8% for DMSO versus 96.9 ± 1.6% for larixyl in C57BL/6 males (*n* = 9 for each group) subjected to either DMSO or larixyl 7-day treatment after TBI. These data strongly support the hypothesis that TRPC6 activation may underlie the deleterious effect of TBI on endothelial function.

**Fig. 7:**
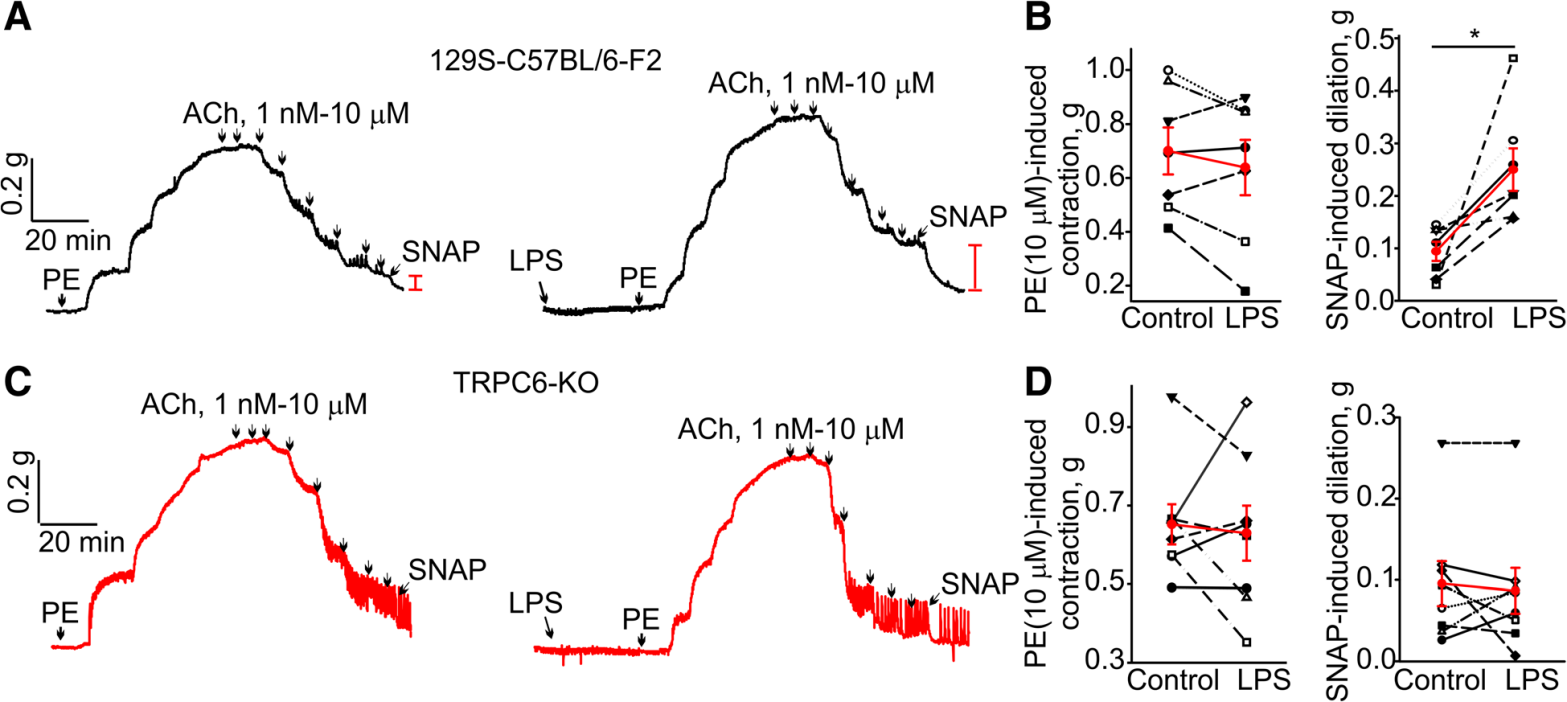
One hour in vitro pre-treatment with LPS induced endothelial dysfunction in 129S-C57BL/6-F2 but not in 129S-C57BL/6-TRPC6 knockout mouse aortic arch rings. **a** The LPS effect in wild type 129S-C57BL/6-F2 mouse aortic arch rings. Sample traces are shown. LPS was added at the time indicated by the arrow in the beginning of the right trace. **b** Summary for the data in **a**. **c**, **d** Sample traces and a summary for the data obtained in aortic arch rings isolated from 129S-C57BL/6-TRPC6 knockout mice. PE - phenylephrine, ACh - acetylcholine, LPS - lipopolysaccharide

To further support this hypothesis, we compared endothelial function in 129S-C57BL/6-F2 aortic arch rings in vitro treated with LPS for 1 h. LPS is known to activate TRPC6 in a TLR4-dependent manner in endothelial cells. To quantify the effects of LPS on endothelial function in the rings, we determined the amplitudes of S-nitroso-*N*-acetyl-D,L-penicillamine (SNAP)-induced dilations in phenylephrine-precontracted aortic rings treated with 10 μM acetylcholine (Fig. 7a and b). SNAP is an NO donor and causes full dilation of the ring due to a direct action of free NO on aortic smooth muscles, while bypassing the endothelium. SNAP treatment provides a measure of total smooth muscle ability to dilate in the presence of NO. We found that before LPS treatment SNAP effects were small in acetylcholine-dilated, phenylephrine-precontracted aortic rings (0.09 ± 0.02 g), whereas SNAP-induced dilation was significantly greater after LPS treatment in the same rings (0.25 ± 0.04 g; *n* = 7). Interestingly, LPS treatment did not alter endothelial function in aortic arch rings from 129S-C57BL/6 TRPC6 knockout mice, again implicating TRPC6 activation in mediating aortic endothelial dysfunction (Fig. 7c and d, SNAP effects: 0.09 ± 0.03 g before LPS treatment versus 0.09 ± 0.02 g after LPS treatment; *n* = 8).

### TLR4 activation is also essential for mediating TBI-associated endothelial dysfunction

**Fig. 8:**
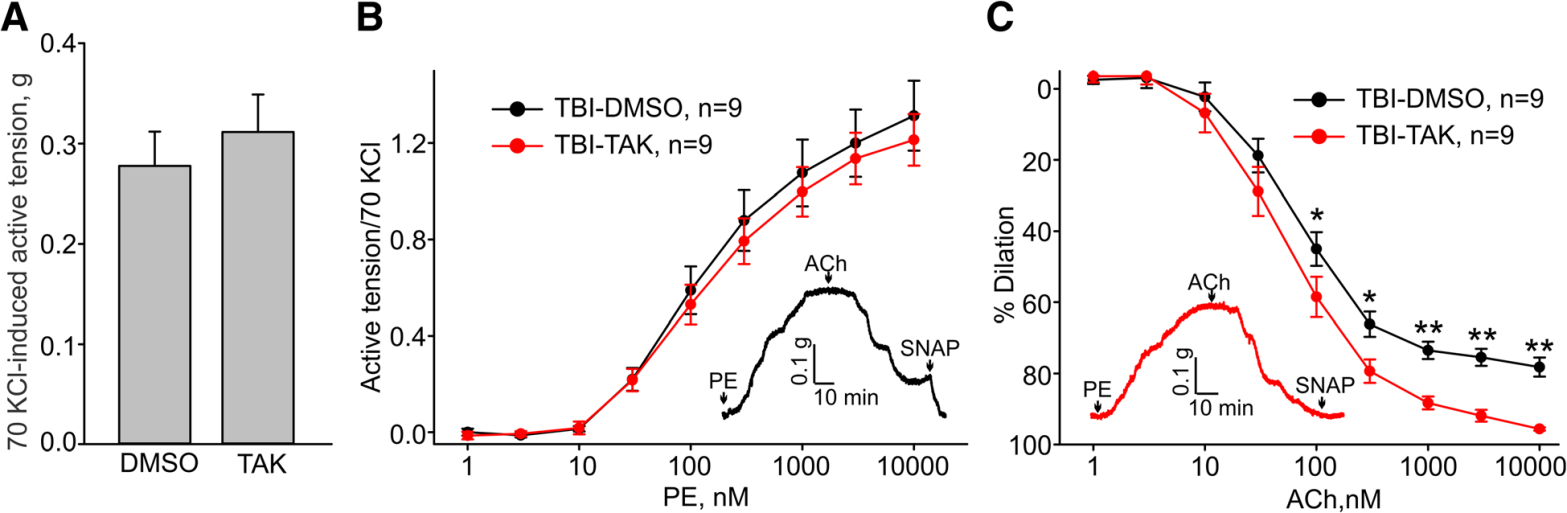
TLR4 inhibitor, TAK-242, treatment decreased TBI-associated aortic endothelial dysfunction in C57BL/6 mice. **a** Summary data for the active tensions measured in the presence of 70 mM KCl in aortic arch rings from TBI C57BL/6 mice treated in vivo with DMSO or TAK-242 via intraperitoneal injection for 7 days. **b** Concentration-response relationships for phenylephrine-induced contractions in aortic arch rings from TBI C57BL/6 mice treated with DMSO or TAK-242 in vivo. **c** Concentration-response relationships for acetylcholine-induced contractions in phenylephrine-precontracted rings from sham and TBI C57BL/6 mice treated with DMSO or TAK-242 in vivo. The insets in **b** and **c** show the sample traces of active tension changes in aortas from the TBI-DMSO group (black lines) and TBI-TAK-242 group (red lines) mice. PE - phenylephrine, ACh - acetylcholine

Tonic TRPC6 activation in the endothelium is TLR4 dependent. Therefore, we next set out to determine whether TLR4 signaling contributes to mediating TBI-associated endothelial dysfunction by performing in vivo treatment with TAK-242, a specific inhibitor of TLR4. We found that the average amplitudes of 70 mM KCl contractions in the aortic rings from mice treated with TAK-242 or the vehicle were not different in the tested groups (0.28 ± 0.03 g, *n* = 9 versus 0.31 ± 0.04 g, *n* = 9 for TBI-DMSO and TBI-TAK-242 groups, respectively, Fig. 8a). The average 10 μM phenylephrine-induced contractions (normalized to the peak amplitudes of 70 mM KCl-induced contractions) exhibited similar amplitudes in those two groups (1.31 ± 0.14, *n* = 7 versus 1.21 ± 0.11, *n* = 7 for TBI-DMSO and TBI-TAK-242 groups, respectively, Fig. 8b). Notably, endothelial function was significantly improved in the TBI-TAK-242 group compared to the TBI-DMSO group (78.2 ± 2.7%, *n* = 9 versus 95.6 ± 0.5%, *n* = 9 for DMSO and TAK-242, respectively, Fig. 8c), supporting the hypothesis that activation of the TBI-TLR4-TRPC6 signaling cascade may be involved in mediating TBI-associated aortic endothelial dysfunction in TBI mice.

### TBI does not affect TRPC6 expression in the endothelial layer of the aortic arch

**Fig. 9:**
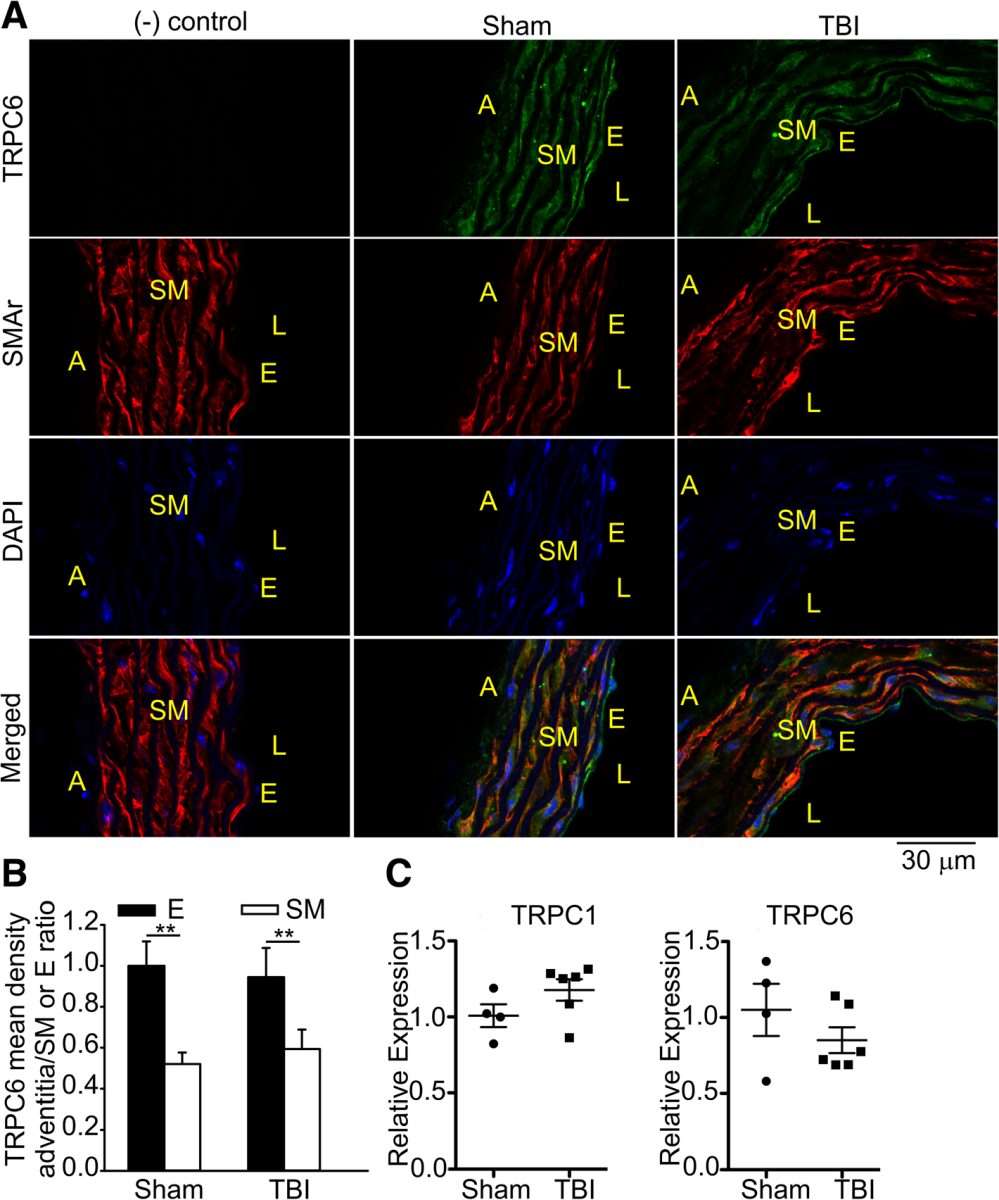
Immunofluorescence and qPCR experiments indicate that TRPC6 expression is not altered by TBI. Immunofluorescence images of the aortic arch sections incubated with or without the TRPC6-antibody (green) and the smooth muscle actin antibody (red) are shown in **a**. Blue color corresponds to the DAPI staining. The aortic arches were isolated from sham or TBI 129S-C57BL/6-F2 mice. A adventitia, SM smooth muscle layer, E endothelium layer, L lumen, × 40 magnification. **b** A summary for data presented in **a**. **c** qRT-PCR was used to measure the expression of TRPC1 and TRPC6 mRNAs, as indicated. Data presented were normalized to an internal control encoding hypoxanthine-guanine phosophoribosyltransferase (Hprt) and are expressed relative to levels in the control sham mice. Relative expression = 2^−ΔΔCt^, where ΔΔCt = (Ct_TBI_ − Ct_Hprt_)–(Ct_sham_ − Ct_Hprt_). Each circle represents an individual mouse. The mean ± S.E. is also indicated

We next examined whether TBI affects TRPC6 expression in the aortic vascular wall using immunofluorescence staining. We found that TRPC6 expression was unaltered in the endothelial layer after TBI (Fig. 9a, sham 1.0 ± 0.12 versus TBI 0.95 ± 0.14, *n* = 3). No primary antibody control was used to confirm the specificity of the TRPC6 antibody. Notably, in both TBI and sham aortic arches, expression of TRPC6 was significantly lower in smooth muscle cell layer as compared to the endothelium (*p* < 0.01; Fig. 9b, sham 1.0 ± 0.12 for the endothelium versus 0.52 ± 0.06 for smooth muscles; TBI 0.95 ± 0.14 for the endothelium versus 0.59 ± 0.09 for smooth muscles). Similarly, we failed to detect any change in TRPC6 mRNA expression in the aorta following TBI (Fig. 9c).

## Discussion

The growing incidence of TBIs, in addition to increasing evidence of long-term and systemic sequalae, highlights the importance of developing new approaches and treatment paradigms. In this study, we demonstrated that closed-head mild TBI causes long-lasting systemic endothelial dysfunction, which is resolved between 7 and 21 day post-injury, in mice. We found that TRPC6 activation may underlie the development of this systemic endothelial dysfunction after TBI. Furthermore, we established that larixyl acetate, an inhibitor of TRPC6, is effective in preventing TBI-associated systemic endothelial dysfunction when it was administered intraperitoneally for 7 days following TBI.

Our finding that closed-head mild TBI-induced aortic endothelial dysfunction in mice of three different genetic backgrounds (C57BL/6, 129S, and 129S-C57BL/6-F2) indicates that this is not a limited phenomenon and is consistent with a recent study demonstrating that severe open-head TBI may cause microvascular endothelial dysfunction in the mesenteric bed [18]. In this previous study, it was proposed that vessels of the systemic circulation may have a “molecular memory” of neurotrauma that may continue for 24 h. We have extended these studies and shown that a mild closed-head TBI also causes endothelial dysfunction of systemic conduit vessels, such as the aorta and we provide evidence that the TBI-associated systemic vascular dysfunction lasts at least 7 days post-injury.

The open-head TBI-associated endothelial dysfunction in mesenteric arteries previously reported, was suggested to occur due to upregulation of arginase, a Mn^2+^-containing metalloenzyme that converts L-arginine to urea and ornithine [37, 38]. As L-arginine is the substrate for endothelial NO synthase (eNOS), arginase-induced degradation of L-arginine limits eNOS-dependent NO production and subsequent vasodilation. In our studies in the aorta following mild closed-head TBI, we found that TRPC6 genetic ablation prevents TBI-associated aortic endothelial dysfunction. As TRPC6 is a Ca^2+^ permeable channel, and intracellular Ca^2+^ rises may rapidly increase arginase expression [39], it is possible that TRPC6 is an upstream element that contributes to increasing arginase expression. However, we cannot rule out that different molecular mechanisms may underlie endothelial dysfunction in the aorta and mesenteric arteries. Future experiments will be required to determine whether there is a difference between the aortic and mesenteric beds in regard to arginase expression and activity.

During mild TBI, blood-brain barrier disruption allows the release of a plethora of pro-inflammatory and damage-associated molecules into the systemic circulation [40]. Increased levels of chemokine CCL2; TNF-α; and interleukins (IL-) 6, 8, and 10 have previously been found in blood serum several days after severe TBI [41, 42]. However, it remains unclear which molecule, or factor, triggers TRPC6 activity after mild TBI in our mouse model. Notably, we did not observe any increase of TRPC6 expression levels in the aortic wall after TBI indicating that changes in TRPC6 activity rather than expression may be mediating aortic endothelial dysfunction. Consistent with this, we found that activation of TLR4 signaling via LPS caused pronounced aortic endothelial dysfunction without TBI, in a TRPC6-dependent manner. We propose that a TLR4 agonist may be present in the systemic circulation after TBI that triggers TRPC6 activity. This hypothesis is supported by a previous report that TLR4 genetic ablation reduces tissues injury events associated with brain trauma [43] and by our data indicating that 7-day treatment with TAK-242, a specific inhibitor of TLR4, significantly improved endothelial function in TBI mice (Fig. 8).

A recent study demonstrated that TBI is also associated with significant cardiac dysfunction identified by decreased left ventricular ejection fraction and fractional shortening, which was observed up to 30 days post-TBI [44]. The authors established that splenectomy significantly decreased cardiac dysfunction, but not neurological or cognitive function, after TBI. Thus, it appears that the TBI-induced neuroinflammation may trigger systemic inflammation and immune cell infiltration in the cardiac tissue, further implicating systemic immune response as a factor underlying TBI-mediated cardiovascular dysfunction.

In this study, we found that the genetic ablation of TRPC1 in mice does not protect from TBI-associated vascular dysfunction. These data are consistent with the observation by Peters et al. [45] that TRPC1−/− mice exhibited similar neurological deficits as wild type mice up to 21 days after closed-head mild TBI. Interestingly, Peters et al. [45] found that carvacrol, 5-isopropyl-2-methylphenol, isolated from the essential oil of *Origanum vulgare*, was effective in improving neurological severity score in TRPC1−/− mice but not in wild type mice, implicating TRPC1 elimination as a sensitizer for the carvacrol beneficial effect after TBI. However, Peters et al. [45] did not investigate the effect of closed-head TBI on the vasculature.

Importantly, we found that pharmacological inhibition of TRPC6 with larixyl acetate was effective at preventing TBI-induced systemic vascular dysfunction (Fig. 4). Larixyl acetate-dependent inhibition of TRPC6 was first described in the elegant paper by Dr. Michael Schaefer’s research team [36]. Urban et al. [36] demonstrated that the compound’s apparent IC_50_ value was approximately 0.65 μM for TRPC6, which is 10–100× lower than that for other members of the TRPC channel subfamily. Importantly, the authors showed that larixyl acetate’s cytotoxicity was very low and that the compound did not lose its bioactivity even after a 24-h incubation in citrate-supplemented human whole blood at 37 °C. Together, these data suggest that larixyl acetate treatment may be an effective therapeutic strategy for limiting the damaging effects on TBI on the systemic vasculature.

## Conclusions

We show for the first time that TRPC6 genetic ablation or pharmacological inhibition with larixyl acetate prevents TBI-associated systemic endothelial dysfunction in mice. These findings identify TRPC6 as a promising target for developing new therapeutic drugs to treat endothelial dysfunction after TBI.

## Abbreviations

ACh: Acetylcholine
Hprt: Hypoxanthine-guanine phosophoribosyltransferase
LPS: Lipopolysaccharide
NO: Nitric oxide
PE: Phenylephrine
SNAP: S-nitroso-*N*-acetyl-D,L-penicillamine
TBI: Traumatic brain injury
TLR4: Toll-like receptor type 4
TRPC: Transient receptor potential canonical
